# User-Centered Design of a Web-Based Tool to Support Management of Chemotherapy-Related Toxicities in Cancer Patients

**DOI:** 10.2196/jmir.9958

**Published:** 2019-03-28

**Authors:** Rebecca M Prince, Anthony Soung Yee, Laura Parente, Katherine A Enright, Eva Grunfeld, Melanie Powis, Amna Husain, Sonal Gandhi, Monika K Krzyzanowska

**Affiliations:** 1 University Health Network Toronto, ON Canada; 2 University of Toronto Toronto, ON Canada; 3 Trillium Health Partners Mississauga, ON Canada; 4 Ontario Institute for Cancer Research Toronto, ON Canada; 5 Sunnybrook Odette Cancer Centre Toronto, ON Canada

**Keywords:** prototype, Web-based tool, toxicity management, chemotherapy, self-management

## Abstract

**Background:**

Cancer patients receiving chemotherapy have high symptom needs that can negatively impact quality of life and result in high rates of unplanned acute care visits. Remote monitoring tools may improve symptom management in this patient population.

**Objective:**

This study aimed to design a prototype tool to facilitate remote management of chemotherapy-related toxicities.

**Methods:**

User needs were assessed using a participatory, user-centered design methodology that included field observation, interviews, and focus groups, and then analyzed using affinity diagramming. Participants included oncology patients, caregivers, and health care providers (HCPs) including medical oncologists, oncology nurses, primary care physicians, and pharmacists in Ontario, Canada. Overarching themes informed development of a Web-based prototype, which was further refined over 2 rounds of usability testing with end users.

**Results:**

Overarching themes were derived from needs assessments, which included 14 patients, 1 caregiver, and 12 HCPs. Themes common to both patients and HCPs included gaps and barriers in current systems, need for decision aids, improved communication and options in care delivery, secure access to credible and timely information, and integration into existing systems. In addition, patients identified missed opportunities, care not meeting their needs, feeling overwhelmed and anxious, and wanting to be more empowered. HCPs identified accountability for patient management as an issue. These themes informed development of a Web-based prototype (*bridges*), which included toxicity tracking, self-management advice, and HCP communication functionalities. Usability testing with 11 patients and 11 HCPs was generally positive; however, identified challenges included tool integration into existing workflows, need for standardized toxicity self-management advice, issues of privacy and consent, and patient-tailored information.

**Conclusions:**

Web-based tools integrating just-in-time self-management advice and HCP support into routine care may address gaps in systems for managing chemotherapy-related toxicities. Attention to the integration of new electronic tools into self-care by patients and practice was a strong theme for both patients and HCP participants and is a key issue that needs to be addressed for wide-scale adoption.

## Introduction

Cancer patients receiving chemotherapy have high symptom burden, which is reflected in their frequent utilization of the emergency department (ED) and high rates of hospitalization during treatment [1-4]. Chemotherapy-related toxicities usually occur between ambulatory visits to the oncology clinic. Unplanned acute care visits among patients receiving systemic therapy in routine practice are likely a reflection of suboptimal management of these toxicities due to inadequate use of preventative strategies or lack of timely access to advice and assessment by the health care team. Some ED visits and hospitalizations may be potentially avoidable with proactive monitoring between clinic visits [5]. The widespread diffusion of health information technology (HIT) represents an opportunity to address gaps in current health care systems with access to Web-enabled devices becoming increasingly common.

Technological solutions have been found to be acceptable to a wide range of populations including older individuals and those with little experience using Web-based technologies [5-8]. Web-based solutions allow an immediacy of access to information and feedback that paper-based systems are not able to provide [7,9] and with a greater degree of accuracy [10]. The design of an effective Web-based chemotherapy toxicity management tool requires an understanding of factors associated with the interaction between humans, technology, and care context to ensure uptake by end users and integration into existing clinical workflows [11]. Engaging end users from the outset also increases external validity and results in highly accurate and relevant solutions while avoiding features and functionalities that were not relevant or useful in existing tools.

To design a prototype Web-based tool to facilitate remote management of chemotherapy-related toxicities, we used an iterative, participatory design methodology informed by human factors principles. As the majority of side effects and subsequent ED visits and hospitalizations occur between clinic visits, we focused on the needs of patients receiving chemotherapy in the outpatient setting. We initiated this study at a time when the evidence base regarding the development and effectiveness of such tools was limited; hence, we felt a local solution was needed [12-14]. A recently published single institution randomized trial of electronic symptom tracking between clinic visits in patients with advanced cancer receiving chemotherapy has shown improvement in patient outcomes including fewer ED visits and hospitalizations [15,16]. However, HIT is an area that is evolving rapidly and, as such, tools which began development over a decade ago [12] may no longer be as relevant. Furthermore, existing tools have mostly undergone academic development in a limited number of settings and have not yet moved into routine practice in the cancer context so there were no *off the shelf tools* that could be easily adopted. In addition, none of the existing tools have been validated in Canada, so it is unclear whether they would be relevant to the Canadian context of a universal health care system that includes provincially organized but locally delivered cancer care. Not all publicly funded health systems have a similar organization, availability of resources, and result in patient populations with different needs. Successful implementation of an electronic tool requires deep understanding of the required features and functionalities and of the health system into which it will be deployed but can also provide insights applicable beyond local context, thus adding to the growing body of knowledge.

## Methods

### Study Overview

To design a prototype of an electronic tool to address gaps in chemotherapy-related symptom management, we used a user-centered participatory design methodology [17]. A Web-based tool was preferred to allow use on any internet-enabled device to mitigate logistical and cost concerns. Potential end users, including patients, their caregivers, and health care providers (HCPs; medical oncologists, primary care physicians, and oncology nurses and pharmacists) were involved in all steps of prototype conceptualization and development. The study was approved by the University Health Network and Trillium Health Partners ethics boards. Informed consent was obtained from all participants; all participants received an honorarium for their time.

### Recruitment and Questionnaire Administration

A convenience sampling approach was utilized whereby HCPs were invited to participate directly by a study team member. Patients and their caregivers were invited to participate by their treating medical oncologist or through an email sent out by the Cancer Care Ontario Patient and Family Advisory Council. Patient participants were required to have received chemotherapy for any cancer type with any intent within the previous 2 years. To ensure a wide sampling of views, each participant undertook only one study activity (ethnographic field study, focus group, or prototype testing). All participants completed a baseline questionnaire to assess their level of interaction with information technology. The patient questionnaire consisted of 11 demographic questions and 10 information technology questions; the HCP questionnaire consisted of 11 demographic questions and 11 information technology questions. For questions estimating hours of computer and internet usage per day, the lower range number was used for analysis.

### User Needs Assessment

To understand and gather insights into the context in which care is provided, we utilized the event-focused ethnographic field study methodology of Bloomberg et al [17] consisting of field observation and interviews and focus groups with potential end users. To understand the tasks, workflows, information requirements and usage patterns, communication and decision-making mechanisms, and use of supporting systems involved in toxicity management, field observations were undertaken. Field observation consisted of silent ﬁrst-hand observation [18] of interactions between HCPs and patients during routine clinic visits in the ambulatory clinical setting with contemporaneous notes taken by 2 2 human factors specialists (ASY and LP). Following each field observation session, separate semistructured interviews were held with providers and patients to gather further information on gaps and facilitators. Interviews were guided by a script but left room to clarify any observed issues to gain deeper insights. Contemporaneous notes were taken, and interviews were audio recorded. Saturation of patient insights, defined as exhaustion of new feedback and themes [19], was not reached following the first round of ethnography; so additional patients were recruited and studied until saturation was reached, as confirmed by sampling of 2 additional patients. A total of 5 study team members confirmed saturation by consensus.

To encourage open discussion of views on issues and to minimize the risk of power imbalance, separate HCP and patient and caregiver focus groups were held and moderated by 2 human factors specialists (ASY and LP). Each participant attended a 1- to 2-hour focus group. Focus groups utilized open-ended questions and probes to generate discussion about gaps in the current health system related to symptom management as well as the content and functionality of a toxicity management tool. Focus groups were audio recorded and contemporaneous notes were taken.

### Analysis

Findings from demographics and preferences questionnaires were summarized using descriptive statistics. Qualitative data gathered during the ethnographic field studies and focus groups were thematically analyzed using the affinity diagramming method of Holtzblatt and Beyer [20] during ideation sessions.

Preparation for the affinity diagramming method consisted of a coding exercise on all the gathered data. Moreover, each of the 2 human factors specialists (ASY and LP) reviewed all data from all sessions generating codes denoting relevant keywords, phrases, and quotes from participant data. Each source of data (field observation and interviews and focus groups) was analyzed separately, which afforded methodological triangulation [21].

These codes were used in the ideation sessions, which were attended by 2 human factors study members (ASY and LP) and 3 HCP study members (RP, MK, and MP). On the basis of the principles of a Team Interpretation session [20], the 5 study members discussed each piece of coded data, sharing insights from the clinical and human factors perspectives. Through discussion of coded data, natural groupings began to form, which developed into themes that encapsulated each natural grouping. For example, one quote from an HCP, “Patients are overwhelmed. My guess is that 95% of the information is instantly forgotten.” was coded as *Overwhelmed* and grouped under the patient-specific theme of *Anxiety and feeling overwhelmed*.

Consensus on the themes was reached by all 5 members, and theme saturation was achieved when no new themes emerged for any presented piece of coded data [19]. This method allowed for effective identification of interrelated factors and existing issues related to remote management of chemotherapy-related toxicities; these themes informed the design of the prototype Web-based toxicity management tool.

### Design and Usability Testing

On the basis of the different functional and information requirements gathered during the needs assessment, distinct interactive prototype interfaces were designed for both the patient and HCP roles. Usability testing was facilitated by human factors specialists and comprised hour-long cognitive walk-throughs, where participants performed realistic tasks while *thinking aloud* [22]. Depending on participant preference, usability testing was conducted either in a private room in the clinical area or at the human factors usability laboratories. The human factors specialists (ASY and LP) explained the purpose and objectives of the usability testing to participants and presented the low fidelity prototype (screen shots without any of the interactive functionality) with scenarios for the participant to complete using the prototype. Sessions were observed, videotaped, and audio taped, and contemporaneous notes were taken. Participants were debriefed at the conclusion of testing to ascertain if any further issues arose during testing.

Target recruitment for usability testing was 4 to 5 participants in the patient and caregiver and clinician groups, respectively, to identify up to 80% of usability problems as recommended in the literature [11]. The tool, comprising both a patient-facing and clinician-facing interface, was refined over 2 rounds of usability testing, resulting in a higher fidelity interactive prototype.

The data from the patient and caregiver and clinician groups were analyzed separately. The data from the video recordings from usability testing also underwent thematic analysis using affinity diagramming methodology [20] with 2 human factors specialists (ASY and LP) reaching consensus on the issues on both interfaces discovered through usability testing. The 2 human factors specialists jointly rank ordered the issues using a weighted decision matrix [23], assigning weights and ratings to various criteria including *Ease of design change*, *Severity of usability issue*, *Benefit to patient*, and *Benefit to HCP*, for each of the identified issues. The issues were iterated upon in the prototype starting from the highest overall rank until all issues were addressed.

## Results

### Participant Demographics

**Figure 1:**
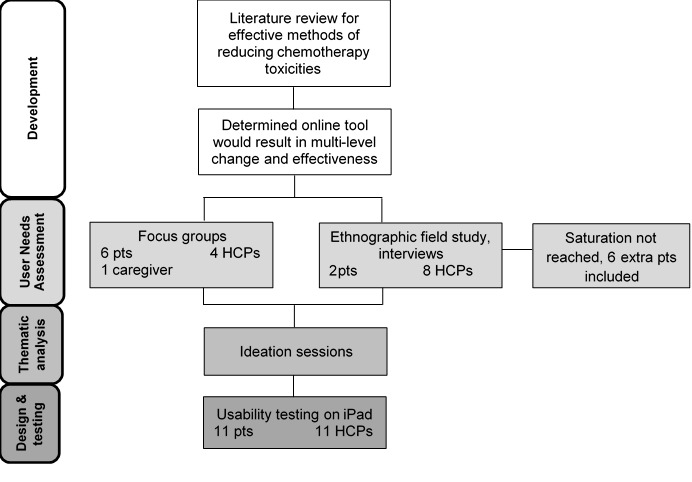
Flow diagram of study design. HCP: health care providers; pts: points.

Between December 2014 and November 2015, 49 patients, caregivers, and HCPs participated in the study. Overall, 8 patients and 8 HCPs participated in ethnographic field studies; 6 patients, 1 caregiver, and 4 HCPs participated in focus groups; and 11 patients and 11 HCPs participated in usability testing (Figure 1). Baseline characteristics of study participants are shown in Table 1; as only 1 caregiver participated, findings were combined with patient responses. Patient and caregiver participants were primarily female (58% [15/26]) with a median age of 55 years, had a diagnosis or cared for someone with a diagnosis of breast cancer (31% [8/26]), had at least a college education (81% [21/26]), and spoke English as their first language (92% [24/26]). HCPs were primarily female (80% [16/20]) with a median age of 50 years, had been in the medical profession on average for 20 years, spoke English as their first language (85% [17/20]), and had a hospital-based practice (90% [18/20]).

**Table 1:** Summary of participant demographics and clinical characteristics.

Characteristics		All (n=46)	Patients and caregivers (n=26)	Health care providers (n=20^a^)
**Gender, n (%)**				
	Male	13 (28)	10 (38)	3 (15)
	Female	31 (67)	15 (58)	16 (80)
	Missing	2 (4)	1 (4)	1 (5)
**Age (years)**				
	Median (range)	—^b^	55 (29-75)	50 (31-65)
	Missing	—	1	1
**Profession, n (%)**				
	Family physician	—	—	2 (10)
	Medical oncologist	—	—	5 (25)
	Oncology nurse	—	—	12 (60)
	Pharmacist	—	—	1 (5)
**Years in health care**				
	Median (range)	—	—	20 (1.5-41)
	Missing	—	—	1
**Diagnosis, n (%)**				
	Gastrointestinal cancer	—	5 (19)	—
	Breast cancer	—	8 (31)	—
	Lung cancer	—	2 (8)	—
	Lymphoma	—	6 (23)	—
	Other	—	2 (8)	—
	Missing	—	2 (8)	—
**Treatment, n (%)**				
	Chemotherapy	—	25 (96)	—
	Radiation	—	13 (50)	—
	Surgery	—	13 (50)	—
**Education, n (%)**				
	Professional/ graduate degree	—	8 (31)	—
	College/university	—	13 (50)	—
	High school	—	3 (12)	—
	Primary/middle school	—	2 (8)	—
**Income, n (%)**				
	CAN $30-59k	—	3 (12)	—
	CAN $60-89k	—	5 (19)	—
	>CAN $90k	—	12 (46)	—
	Prefer not to say	—	6 (23)	—
**First language, n (%)**				
	English	41 (89)	24 (92)	17 (85)
	Other	5 (11)	2 (8)	3 (5)
**Practice setting, n (%)**				
	Community-based clinic	—	—	2 (10)
	Hospital	—	—	7 (35)
	Hospital clinic	—	—	11 (55)

^a^Data missing for 3 health care providers.

^b^Not applicable.

### Baseline Technology Use

Computer and internet use were very common among HCPs, patients, and caregivers (Table 2) with the majority of participants using computers at work (80% [37/46]) and at home (93% [43/46]) and having access to internet at home (98% [45/46]). HCPs reported using electronic devices approximately twice as much as patients (6.5 vs 3.5 hours per day). Most participants reported being *comfortable* or *very comfortable* using computers, smartphones and tablets, internet, email, and instant messaging. Only 50% (23/46) of patients and HCPs felt *comfortable* using social media with 50% (13/26) of patients and 15% (3/20) of HCPs either *not using* or feeling *not at all comfortable* with social media platforms.

### User Needs

**Figure 2:**
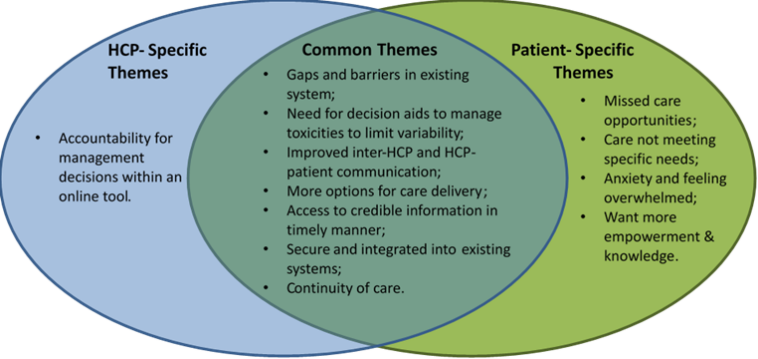
Summary of findings from thematic analysis of user needs data. HCP: health care providers.

Thematic analysis of data collected from the ethnographic field studies and focus groups revealed themes of the recognition of gaps in the current health system, the existence of barriers to accessing care, and the need for timely support in decision making, which were common to both patients and HCPs (Figure 2). Additional themes were specific to either patients and caregivers or to HCPs. Patients and caregivers recognized that health systems often do not match their needs and that many psychological symptoms are not well addressed, especially feelings of being overwhelmed and anxious. HCPs identified the need for clear lines of accountability for any decisions and advice given to patients through a Web-based tool.

### Design of Interactive Prototype

**Figure 3:**
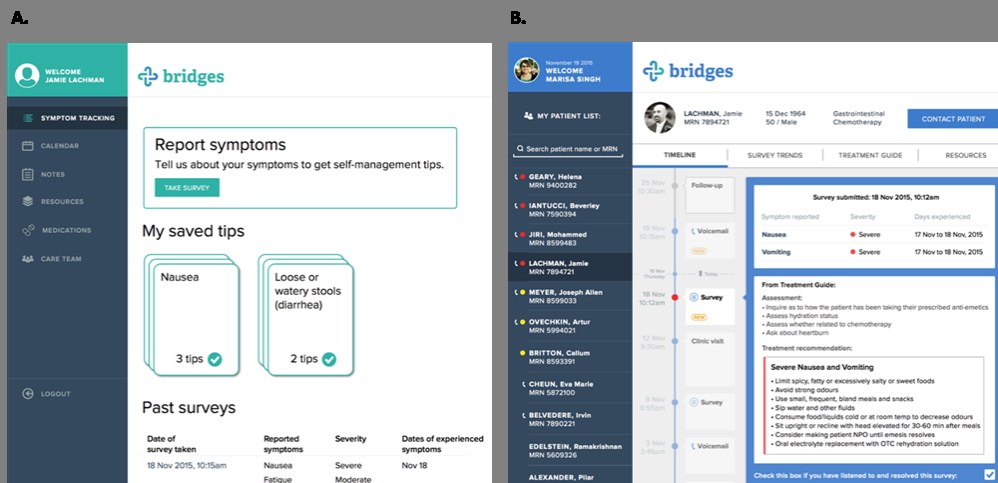
Select screenshots of bridges from the patient/caregiver (panel A) and health care provider (panel B) user interfaces.

The design of an interactive prototype with separate patient and HCP interfaces, *bridges*, was informed by the findings of the user needs assessment. The patient interface includes functionality for toxicity reporting, self-management advice based on the reported toxicities, an appointment calendar, educational materials, and options for HCP communication (Figure 3). The HCP interface includes functionality for viewing patient visit history and voicemails left by the patient, trending of toxicity reporting data, and a standardized treatment guide for handling common chemotherapy-related toxicities.

### Usability Testing

Iterative rounds of usability testing were undertaken with 22 participants (11 patients and 11 HCPs) to further refine the prototype and evaluate end-user needs. During usability testing, patients noted that it is often left to them to self-organize their care including integration of complex scheduling information and medication administration (Textbox 1). Many patients desired access to an up-to-date Web-based calendar from the hospital or a calendar they could synchronize with their own device. Patients were very clear that they wanted access to information specific to their cancer type and treatment regimen rather than general information which may not apply to them. They had concerns about whether they would actually use the tool if they were feeling unwell and highlighted that the ability for a caregiver to fill it out for them would be useful. Patients also provided insight into the fact that care teams are frequently comprised of many members that change regularly.

Issues noted by HCPs included concerns of how a Web-based tool would be integrated into existing workflows and procedures, especially existing electronic medical records, to avoid entering the same information into multiple HIT systems. The context within which patient-reported toxicities occurred was felt to be very important, so the ability to add notes within the app was desired. Issues of confidentiality and privacy were highlighted as critical and requiring clarification, particularly with regard to patient consent around communication between HCPs and caregivers. The ability to prioritize patient symptoms and other issues in order of urgency was also felt to be important to ensure that the most serious issues were addressed first. The use of a treatment guide for symptom management was seen as a useful tool to ensure HCPs give consistent advice to patients.

**Table 2:** Baseline computer and information technology use.

Category and Response		All (n=46)	Patients and caregivers (n=26)	Health care providers (n=20^a^)
**Computer use at work, n (%)**				
	Yes	37 (80)	17 (65)	20 (100)
	No	7 (15)	7 (27)	0 (0)
	Missing	2 (4)	2 (8)	0 (0)
**Computer use at home, n (%)**				
	Yes	43 (93)	24 (92)	19 (95)
	No	3 (7)	2 (8)	1 (5)
**Internet access at home, n (%)**				
	Yes	45 (98)	25 (96)	20 (100)
	No	0 (0)	0 (0)	0 (0)
	Missing	1 (2)	1 (4)	0 (0)
Hours on computer, smartphone, and tablet, per day, median (range)		—^b^	3.5 (<1-9)	6.5 (1-12)
**Comfort using a computer, n (%)**				
	Not at all	1 (2)	1 (4)	0 (0)
	A little	2 (4)	1 (4)	1 (5)
	Comfortable	15 (33)	6 (23)	9 (45)
	Very comfortable	27 (59)	17 (65)	10 (50)
	Missing	1 (2)	1 (4)	0 (0)
**Comfort using a smartphone or tablet, n (%)**				
	Do not use	1 (2)	1 (4)	0 (0)
	Not at all	3 (7)	2 (8)	1 (5)
	A little	4 (9)	1 (4)	3 (15)
	Comfortable	15 (33)	6 (23)	9 (45)
	Very comfortable	23 (50)	16 (62)	7 (35)
Hours on internet per day, median (range)		—	2 (<1-12)	3 (1-12)
**Comfort using internet, n (%)**				
	Do not use	1 (2)	1 (4)	0 (0)
	Not at all	0 (0)	0 (0)	0 (0)
	A little	3 (7)	3 (12)	0 (0)
	Comfortable	15 (33)	5 (19)	10 (50)
	Very comfortable	27 (59)	17 (65)	10 (50)
**Comfort using email, n (%)**				
	Do not use	0 (0)	0 (0)	0 (0)
	Not at all	2 (4)	2 (8)	0 (0)
	A little	1 (2)	1 (4)	0 (0)
	Comfortable	14 (30)	5 (19)	9 (45)
	Very comfortable	29 (63)	18 (69)	11 (55)
**Comfort using instant messaging, n (%)**				
	Do not use	3 (7)	3 (12)	0 (0)
	Not at all	1 (2)	1 (4)	0 (0)
	A little	4 (9)	1 (4)	3 (15)
	Comfortable	12 (26)	4 (15)	8 (40)
	Very comfortable	26 (57)	17 (65)	9 (45)
**Comfort using social media, n (%)**				
	Do not use	11 (24)	9 (35)	2 (10)
	Not at all	5 (11)	4 (15)	1 (5)
	A little	7 (15)	0 (0)	7 (35)
	Comfortable	12 (26)	7 (27)	5 (25)
	Very comfortable	11 (24)	6 (23)	5 (25)
**Technology used in normal workday, n (%)**				
	Computer	—	—	20 (100)
	Smartphone	—	—	11 (55)
	Tablet	—	—	3 (15)

^a^Data missing for 3 health care providers.

^b^Not applicable.

**Patient and health care provider (HCP) themes from usability testing.**

Patients

- Responsible for self-organizing care
- Want appointment calendar synced with hospital
- Want information specific to their cancer and treatment
- Practicalities of using online application—if unwell, want caregivers to fill out
- Heath care is dynamic—care teams often large and ever-changing

Health care providers

- Integration into existing work flows and practices
- Provision of context to patient feedback as notes
- Privacy and consent issues—sharing patient information, especially with caregivers
- Prioritizing incoming information—deal with most serious problems first
- Consistent assessments and provision of standardized management information (ie, treatment guidelines)

## Discussion

### Principal Findings

In our study, patients, caregivers, and HCPs engaged in designing an interactive Web-based prototype of a chemotherapy-related toxicity management tool. Issues within the existing systems identified by participants included the need for HCP-supported decision making and self-management strategies for patients, access to credible and timely information, improved communication (both between patients and HCPs, and among HCPs), and integration of the tool within existing workflows to prevent redundancy and confidentiality issues. Understanding local context including the required features and functionalities and potential implementation issues particular to the local context is essential for long-term success. Our study identified a number of additional functionalities and implementation challenges that add to this growing body of literature such as concerns from providers regarding accountability within the tool.

### Comparison With Previous Work

Functionalities of existing tools for patients undergoing cancer treatment that have been developed and pilot tested range from symptom tracking alone [24-30], symptom tracking in conjunction with self-care support [31-36], and symptom tracking with active symptom monitoring [12,15,37-47]. Our findings confirm previous work, some of which was published before and some during the conduct of our study, such as high levels of patient satisfaction with remote symptom monitoring systems and the need for ease of use of the tool. When using any tool designed to improve the quality of patient care, the local context is a critical factor that must be addressed, as it will determine the success of implementation. Our study also identified additional functionalities and implementation challenges that add to this growing body of literature including the need to streamline etool integration into patient self-care and current clinical practice workflows before wide-scale adoption and accountability for provider actions within the etool necessitating clearly defined roles and responsibilities of users. This complements findings from a previous study by Mooney et al [48] utilizing a telephone-based remote monitoring system for cancer patients that highlighted the need to explicitly address who is going to address alerts, as assuming providers will add these duties on to their existing responsibilities does not work.

The evidence for positive impact of etools for symptom monitoring during cancer treatment is growing. A recent study by Denis et al [46] of Web-based follow-up of self-reported symptoms in lung cancer patients following initial treatment reported improved overall survival. Basch et al [15,16] found that remote monitoring of patient symptoms during chemotherapy resulted in significantly improved quality of life, fewer ED visits and hospitalizations, and was associated with prolonged survival. Both interventions included health team alerting functionality for severe symptoms. Likewise, Chumbler et al [49] found a significant reduction in clinic visits and chemotherapy-related hospitalizations using a Cancer Care Coordination Home Telehealth approach.

### Limitations

Our findings need to be evaluated in the context of study limitations. Our participants were from 2 large urban centers in southern Ontario and, thus, may not represent end-user needs from other jurisdictions. Selection bias may be present as HCPs who volunteered to participate may have been inherently more interested in using HIT solutions. Likewise, patients who contacted the study coordinator directly may represent a more motivated population than a random sample and may not be representative of the views of the overall population. HCP and patient participants were mostly female (HCPs: 16 vs 3; patients/caregivers: 15 vs 10), so findings may not adequately represent the views of their male counterparts. Our patient and caregiver participants had a median age of 55 and reported having at least a college education (81% [21/26]), English as their first language (92% [24/26]), and comfort using the internet and technology. As such, additional work is needed to better understand the needs of end users who are older, non-English speaking, less educated, or less familiar with technology. The design of our interactive prototype is a first step in building an effective electronic tool for patient care. Additional studies are needed to explore whether the tool impacts patient outcomes; although, the recently published trials from the United States [15] and France [46] provide important information regarding the benefits of such systems for patients in controlled settings. Data on wide-scale adoption of etools in this setting are not yet available.

The method of affinity diagramming aims to bring together issues and insights from various stakeholders, from which overarching themes emerge. Although the data were collected using a number of approaches to understand the complexities and subtleties of toxicity management within the Canadian context, it could be argued that the resulting artifact from affinity diagraming affords only a thin description of toxicity management, that is, a limitation of the method is that the resulting affinity diagram is only a brief summary of the themes that emerged, lacking rich context. However, it should be noted that the human factors study members who conducted the ethnographic field study and participated in ideation sessions were also those who designed the prototype. Thus, although the themes from affinity diagramming informed the design of the prototypes, the human factors study members were also able to draw from the contextual data that they experienced firsthand. Although the number of participants required for usability testing has been well-established [11], there is little consensus on the number of participants required for interviews and focus groups [21,50,51]. Given this limitation, the authors instead strived for saturation of the collected data and resulting themes [21] and triangulation using multiple sources of data (field observation, interviews, and focus groups) [51] to arrive at a comprehensive understanding of toxicity management from various stakeholders.

### Conclusions

We have shown that using a human factors design approach for a Web-based application to support management of chemotherapy-related toxicities has the potential to address gaps in cancer care. As these gaps were identified within the local context of care, the design, iterated upon through prototyping and usability testing, seeks to address these needs directly. Our study highlighted that operationalizing a Web-based tool has significant system implications, including assigning responsibility for monitoring the tool to appropriate HCPs and the need to embed the tool into existing workflows and systems. Integration of new etools into self-care by patients and practice is a key issue that needs to be addressed for wide-scale adoption.

## Abbreviations

ED: emergency department
HCP: health care provider
HIT: health information technology

## References

[1] Prince RM, Atenafu EG, Krzyzanowska MK (2015). Hospitalizations during systemic therapy for metastatic lung cancer: a systematic review of real world vs clinical trial outcomes. JAMA Oncol.

[2] Hassett MJ, O'Malley AJ, Pakes JR, Newhouse JP, Earle CC (2006). Frequency and cost of chemotherapy-related serious adverse effects in a population sample of women with breast cancer. J Natl Cancer Inst.

[3] (2018). Cancer Quality Council of Ontario.

[4] Prince RM, Powis M, Zer A, Atenafu EG, Krzyzanowska MK (2019). Hospitalisations and emergency department visits in cancer patients receiving systemic therapy: Systematic review and meta-analysis. Eur J Cancer Care (Engl).

[5] Given CW, Bradley C, You M, Sikorskii A, Given B (2010). Costs of novel symptom management interventions and their impact on hospitalizations. J Pain Symptom Manage.

[6] Carlson LE, Speca M, Hagen N, Taenzer P (2001). Computerized quality-of-life screening in a cancer pain clinic. J Palliat Care.

[7] Gaertner J, Elsner F, Pollmann-Dahmen K, Radbruch L, Sabatowski R (2004). Electronic pain diary: a randomized crossover study. J Pain Symptom Manage.

[8] Mullen KH, Berry DL, Zierler BK (2004). Computerized symptom and quality-of-life assessment for patients with cancer part II: acceptability and usability. Oncol Nurs Forum.

[9] Jamison RN, Raymond SA, Levine JG, Slawsby EA, Nedeljkovic SS, Katz NP (2001). Electronic diaries for monitoring chronic pain: 1-year validation study. Pain.

[10] Stone AA, Shiffman S, Schwartz JE, Broderick JE, Hufford MR (2002). Patient non-compliance with paper diaries. BMJ.

[11] Kushniruk AW, Patel VL (2004). Cognitive and usability engineering methods for the evaluation of clinical information systems. J Biomed Inform.

[12] Kearney N, McCann L, Norrie J, Taylor L, Gray P, McGee-Lennon M, Sage M, Miller M, Maguire R (2009). Evaluation of a mobile phone-based, advanced symptom management system (ASyMS) in the management of chemotherapy-related toxicity. Support Care Cancer.

[13] (2015). Apple.

[14] Børøsund E, Cvancarova M, Moore SM, Ekstedt M, Ruland CM (2014). Comparing effects in regular practice of e-communication and web-based self-management support among breast cancer patients: preliminary results from a randomized controlled trial. J Med Internet Res.

[15] Basch E, Deal AM, Kris MG, Scher HI, Hudis CA, Sabbatini P, Rogak L, Bennett AV, Dueck AC, Atkinson TM, Chou JF, Dulko D, Sit L, Barz A, Novotny P, Fruscione M, Sloan JA, Schrag D (2016). Symptom monitoring with patient-reported outcomes during routine cancer treatment: a randomized controlled trial. J Clin Oncol.

[16] Basch E, Deal AM, Dueck AC, Scher HI, Kris MG, Hudis C, Schrag D (2017). Overall survival results of a trial assessing patient-reported outcomes for symptom monitoring during routine cancer treatment. J Am Med Assoc.

[17] Blomberg J, Giacomi J, Mosher A, Swenton-Wall P (1993). Ethnographic field methods and their relation to design. Participatory Design: Perspectives on Systems Design.

[18] Carayon P, Kianfar S, Li Y, Xie A, Alyousef B, Wooldridge A (2015). A systematic review of mixed methods research on human factors and ergonomics in health care. Appl Ergon.

[19] Rothman M, Burke L, Erickson P, Leidy NK, Patrick DL, Petrie CD (2009). Use of existing patient-reported outcome (PRO) instruments and their modification: the ISPOR Good Research Practices for Evaluating and Documenting Content Validity for the Use of Existing Instruments and Their Modification PRO Task Force Report. Value Health.

[20] Holtzblatt K, Beyer H (2011). Contextual Design. The Encyclopedia of Human-Computer Interaction, 2nd Ed. Soegaard M, Dam RF. editors. The Interaction Design Foundation.

[21] Morse JM (1995). The significance of saturation. Qual Health Res.

[22] Yardley L, Morrison LG, Andreou P, Joseph J, Little P (2010). Understanding reactions to an internet-delivered health-care intervention: accommodating user preferences for information provision. BMC Med Inform Decis Mak.

[23] Wickens CD, Hollands JG, Banbury S, Parasuraman R (2013). Engineering Psychology And Human Performance.

[24] Basch E, Artz D, Dulko D, Scher K, Sabbatini P, Hensley M, Mitra N, Speakman J, McCabe M, Schrag D (2005). Patient online self-reporting of toxicity symptoms during chemotherapy. J Clin Oncol.

[25] Basch E, Artz D, Iasonos A, Speakman J, Shannon K, Lin K, Pun C, Yong H, Fearn P, Barz A, Scher HI, McCabe M, Schrag D (2007). Evaluation of an online platform for cancer patient self-reporting of chemotherapy toxicities. J Am Med Inform Assoc.

[26] Bock M, Moore D, Hwang J, Shumay D, Lawson L, Hamolsky D, Esserman L, Rugo H, Chien AJ, Park J, Munster P, Melisko M (2012). The impact of an electronic health questionnaire on symptom management and behavior reporting for breast cancer survivors. Breast Cancer Res Treat.

[27] Judson TJ, Bennett AV, Rogak LJ, Sit L, Barz A, Kris MG, Hudis CA, Scher HI, Sabattini P, Schrag D, Basch E (2013). Feasibility of long-term patient self-reporting of toxicities from home via the internet during routine chemotherapy. J Clin Oncol.

[28] Macpherson CF, Linder LA, Ameringer S, Erickson J, Stegenga K, Woods NF (2014). Feasibility and acceptability of an iPad application to explore symptom clusters in adolescents and young adults with cancer. Pediatr Blood Cancer.

[29] Snyder CF, Blackford AL, Aaronson NK, Detmar SB, Carducci MA, Brundage MD, Wu AW (2011). Can patient-reported outcome measures identify cancer patients' most bothersome issues?. J Clin Oncol.

[30] Snyder CF, Blackford AL, Wolff AC, Carducci MA, Herman JM, Wu AW, PatientViewpoint Scientific Advisory Board (2013). Feasibility and value of PatientViewpoint: a web system for patient-reported outcomes assessment in clinical practice. Psychooncology.

[31] Berry DL, Hong F, Halpenny B, Partridge A, Fox E, Fann JR, Wolpin S, Lober WB, Bush N, Parvathaneni U, Amtmann D, Ford R (2014). The electronic self report assessment and intervention for cancer: promoting patient verbal reporting of symptom and quality of life issues in a randomized controlled trial. BMC Cancer.

[32] Berry DL, Hong F, Halpenny B, Partridge AH, Fann JR, Wolpin S, Lober WB, Bush NE, Parvathaneni U, Back AL, Amtmann D, Ford R (2014). Electronic self-report assessment for cancer and self-care support: results of a multicenter randomized trial. J Clin Oncol.

[33] Berry DL, Blonquist TM, Patel RA, Halpenny B, McReynolds J (2015). Exposure to a patient-centered, web-based intervention for managing cancer symptom and quality of life issues: impact on symptom distress. J Med Internet Res.

[34] Head BA, Keeney C, Studts JL, Khayat M, Bumpous J, Pfeifer M (2011). Feasibility and acceptance of a telehealth intervention to promote symptom management during treatment for head and neck cancer. J Support Oncol.

[35] Klasnja P, Hartzler A, Powell C, Pratt W (2011). Supporting cancer patients' unanchored health information management with mobile technology. AMIA Annu Symp Proc.

[36] McGee M, Gray P (2016). A handheld chemotherapy symptom management system: results from a preliminary outpatient field trial. Health Informatics J.

[37] Chan MF, Ang E, Duong MC, Chow YL (2013). An online symptom care and management system to monitor and support patients receiving chemotherapy: a pilot study. Int J Nurs Pract.

[38] Chan MF, Ang NK, Cho AA, Chow YL, Taylor B (2014). Online chemotherapy symptom care and patient management system: an evaluative study. Comput Inform Nurs.

[39] Maguire R, McCann L, Miller M, Kearney N (2008). Nurse's perceptions and experiences of using of a mobile-phone-based Advanced Symptom Management System (ASyMS) to monitor and manage chemotherapy-related toxicity. Eur J Oncol Nurs.

[40] Maguire R, Miller M, Sage M, Norrie J, McCann L, Taylor L, Kearney N (2005). Results of a UK based pilot study of a mobile phone based advanced symptom management system (ASyMS) in the remote monitoring of chemotherapy related toxicity. Clin Eff Nurs.

[41] Maguire R, Ream E, Richardson A, Connaghan J, Johnston B, Kotronoulas G, Pedersen V, McPhelim J, Pattison N, Smith A, Webster L, Taylor A, Kearney N (2015). Development of a novel remote patient monitoring system: the advanced symptom management system for radiotherapy to improve the symptom experience of patients with lung cancer receiving radiotherapy. Cancer Nurs.

[42] McCann L, Maguire R, Miller M, Kearney N (2009). Patients' perceptions and experiences of using a mobile phone-based advanced symptom management system (ASyMS) to monitor and manage chemotherapy related toxicity. Eur J Cancer Care (Engl).

[43] Ruland CM, Andersen T, Jeneson A, Moore S, Grimsbø GH, Børøsund E, Ellison MC (2013). Effects of an internet support system to assist cancer patients in reducing symptom distress: a randomized controlled trial. Cancer Nurs.

[44] Weaver A, Love SB, Larsen M, Shanyinde M, Waters R, Grainger L, Shearwood V, Brooks C, Gibson O, Young AM, Tarassenko L (2014). A pilot study: dose adaptation of capecitabine using mobile phone toxicity monitoring-supporting patients in their homes. Support Care Cancer.

[45] Mooney KH, Beck SL, Wong B, Dunson W, Wujcik D, Whisenant M, Donaldson G (2017). Automated home monitoring and management of patient-reported symptoms during chemotherapy: results of the symptom care at home RCT. Cancer Med.

[46] Denis F, Lethrosne C, Pourel N, Molinier O, Pointreau Y, Domont J, Bourgeois H, Senellart H, Trémolières P, Lizée T, Bennouna J, Urban T, El Khouri C, Charron A, Septans A, Balavoine M, Landry S, Solal-Céligny P, Letellier C (2017). Randomized trial comparing a web-mediated follow-up with routine surveillance in lung cancer patients. J Natl Cancer Inst.

[47] Holch P, Warrington L, Bamforth LC, Keding A, Ziegler LE, Absolom K, Hector C, Harley C, Johnson O, Hall G, Morris C, Velikova G (2017). Development of an integrated electronic platform for patient self-report and management of adverse events during cancer treatment. Ann Oncol.

[48] Mooney KH, Beck SL, Friedman RH, Farzanfar R, Wong B (2014). Automated monitoring of symptoms during ambulatory chemotherapy and oncology providers' use of the information: a randomized controlled clinical trial. Support Care Cancer.

[49] Chumbler NR, Kobb R, Harris L, Richardson LC, Darkins A, Sberna M, Dixit N, Ryan P, Donaldson M, Kreps GL (2007). Healthcare utilization among veterans undergoing chemotherapy: the impact of a cancer care coordination/home-telehealth program. J Ambul Care Manage.

[50] Kerr C, Nixon A, Wild D (2010). Assessing and demonstrating data saturation in qualitative inquiry supporting patient-reported outcomes research. Expert Rev Pharmacoecon Outcomes Res.

[51] Fusch P, Ness L (2005). Are We There Yet? Data saturation in qualitative research. The Qualitative Report.

